# Long-Term Follow-Up of Levetiracetam Monotherapy Versus Add-On Therapy in Pediatric Epilepsy

**DOI:** 10.3390/children13080997

**Published:** 2026-07-28

**Authors:** Yukiko Osada, Kosuke Nakano, Masayoshi Nakakuni, Jun Tsuchiya, Madoka Watanabe, Shinji Kobayashi, Miki Akabane, Yoshiaki Yamamoto, Akimasa Yamatani

**Affiliations:** 1 Graduate School of Pharmaceutical Sciences, Meiji Pharmaceutical University, Tokyo 204-8588, Japan; 2 Department of Pharmacy, National Center for Child Health and Development, Tokyo 157-8535, Japan; 3 MSD K.K., Tokyo 102-8667, Japan; 4 Clinical Research Center, National Center for Child Health and Development, Tokyo 157-8535, Japan; 5 H&H CONNECT Co., Ltd., Tokyo 104-0033, Japan; 6 Nature Insight Co., Ltd., Tokyo 101-0021, Japan; 7 Department of Pharmacy, NHO Kanazawa Medical Center, Kanazawa 920-8650, Japan

**Keywords:** epilepsy, pediatric, levetiracetam, monotherapy, add-on therapy, retention rate, irritability, insomnia, database study

## Abstract

**Highlights:**

**What are the main findings?**

- Levetiracetam monotherapy showed a higher treatment retention rate than levetiracetam-containing combination therapy in pediatric patients with epilepsy.
- The rates of irritability or insomnia were similar between the monotherapy and combination therapy groups.

**What are the implications of the main findings?**

- Adding levetiracetam to other anti-seizure medications was not associated with an evident increase in irritability or insomnia.
- These findings may support treatment decisions regarding long-term levetiracetam use in pediatric epilepsy.

**Abstract:**

**Background/Objectives**: Epilepsy treatment often continues long-term from childhood. However, evidence comparing long-term treatment persistence and adverse events (AEs) between levetiracetam (LEV) monotherapy and LEV-containing combination therapy in pediatric patients is limited. This study compared the retention rate and the rate of AEs, including irritability and insomnia, between these treatment strategies. **Methods**: A retrospective cohort study was conducted using data from the Pediatric Medical Information Collection System database. Patients aged ≥0 to <15 years who were diagnosed with epilepsy and prescribed with LEV, based on records registered between 1 April 2016 and 30 September 2025, were included in the study. **Results**: In total, 3576 patients were analyzed. Among them, 2936 were classified under the monotherapy group and 640 under the combination therapy group. The monotherapy group had a significantly higher retention rate than the combination therapy group. The two groups did not significantly differ in terms of the rates of irritability or insomnia. **Conclusions**: In pediatric patients with epilepsy, the retention rate of LEV monotherapy was higher than that of LEV-containing combination therapy. Meanwhile, the rates of irritability or insomnia did not substantially differ between the two groups. Based on these findings, adding LEV to other anti-seizure medications was not associated with an evident increase in the rate of irritability or insomnia. Although anti-seizure medication polytherapy has traditionally been considered to increase the risk of psychiatric and behavioral symptoms, the current study showed a different trend.

## 1. Introduction

In the pharmacological treatment of epilepsy, monotherapy is generally initiated first, with consideration of potential drug interactions and tolerability [1]. If seizures are not adequately controlled with monotherapy, rational combination therapy using agents with different mechanisms of action may be considered [2]. The choice of anti-seizure medication (ASM) is individualized based on seizure type and epilepsy syndrome, taking into account age, sex, comorbidities, medical history, and use of concomitant medications [3].

Approximately 70% of patients with newly diagnosed epilepsy can achieve complete seizure control with appropriate pharmacotherapy [4]. However, some patients managed with multiple ASMs are still challenging to treat. About 39% of patients present with drug-resistant epilepsy, for which dietary therapy or surgery may be considered in addition to polytherapy [5]. In some cases, polytherapy with three or more ASMs is used, and approximately 20% of patients whose seizures have been controlled for at least 1 year receive two to four ASMs [6]. Several ASMs with different mechanisms of action are available. However, reducing or discontinuing medications may worsen seizures in patients receiving multiple ASMs, making regimen simplification difficult in refractory epilepsy [7,8,9,10]. Polytherapy with ASMs may also increase side effects via drug interactions [8]. In children, treatment decisions should further be based on developmental stage and the impact of long-term therapy on daily life and school activities. Therefore, benefit–risk assessment that includes both seizure control and adverse effects is particularly important [11].

LEV is one of the ASMs widely used in clinical practice because it has demonstrated efficacy across a broad range of seizure types and has a low potential for drug interactions [12]. However, this drug is associated with adverse events (AEs), particularly psychiatric and behavioral symptoms, including irritability, aggression, anxiety, anger, and insomnia [13]. Clinical studies have also shown that pediatric patients with epilepsy, similar to adults, may present with nonpsychotic behavioral AEs and insomnia [14,15].

Pharmacological treatment for epilepsy often continues over a long period from childhood. However, limited evidence is available from long-term comparative studies evaluating the retention or AEs rate of LEV monotherapy versus combination therapy including LEV in pediatric patients with epilepsy. Therefore, using data from the Pediatric Medical Information Collection System (P-MICS) database, which is maintained by the National Center for Child Health and Development, the current study compared the retention rate and rate of AEs, including irritability and insomnia, between LEV monotherapy and LEV-containing combination therapy in pediatric patients with epilepsy. In this study, the study outcomes were the retention rate and the rates of irritability and insomnia. The retention rate is an indirect indicator reflecting long-term efficacy and tolerability, and several studies have evaluated the retention rate of LEV in patients with epilepsy [16,17,18]. Irritability is a representative AE of LEV, and insomnia is also a common AE associated with other ASMs [13,19]. Therefore, we investigated whether the rate of these events increases if LEV is used in combination with other ASMs in real-world clinical practice.

## 2. Materials and Methods

### 2.1. P-MICS Database

This study used data from the P-MICS database, which was established by the National Center for Child Health and Development in 2012 as a project supported by the Ministry of Health, Labour and Welfare, with the aim of contributing to drug safety measures and drug development in pediatric populations. The database integrates electronic medical record data and questionnaire information provided by participating medical institutions. Starting in September 2025, electronic medical record data from 11 pediatric hospitals and 32 pediatric clinics across Japan have been accumulated in the database.

### 2.2. Study Design

The current retrospective cohort study compared the retention rate and the rates of AEs, including irritability and insomnia, between LEV monotherapy and combination therapy with LEV and other ASMs in pediatric patients with epilepsy.

This study used data registered in the P-MICS database between 1 April 2016 and 30 September 2025, after excluding patients with missing information on age or sex. The inclusion criteria were as follows: (1) patients diagnosed with epilepsy (the International Classification of Diseases, 10th Revision [ICD-10] code: G40) and prescribed with levetiracetam (Anatomical Therapeutic Chemical [ATC] classification: N03AX14), (2) those aged ≥ 0 to <15 years at the time of epilepsy diagnosis and LEV prescription, and (3) those treated at hospitals. The exclusion criteria were as follows: (1) patients who did not visit the hospital within 100 days after the first prescription of an ASM (ATC classification: N03A), (2) those who had received an antipsychotic drug (ATC classification: N05A) before LEV initiation, and (3) those diagnosed with insomnia (ICD-10 code: G470) and prescribed with hypnotics and sedatives (ATC classification: N05C) before LEV initiation. Patients meeting the eligibility criteria were classified into either the monotherapy or combination therapy groups. The monotherapy group included patients who received LEV as the initial monotherapy. The combination therapy group included patients who received LEV as an add-on therapy during treatment with other ASMs. Considering patient-related circumstances, an allowable prescription gap of up to 14 days was permitted. If the same regimen was re-prescribed within 14 days after interruption, treatment was considered to have continued. The rescue medications listed in Tables S1 and S2 were not regarded as concomitant drugs. However, if the same medication was prescribed for at least 7 consecutive days, it was considered as a concomitant drug.

The pathophysiology and treatment strategies for epilepsy differ according to epilepsy classification. Thus, a subgroup analysis was also conducted among patients with focal epilepsy who met the inclusion and exclusion criteria.

The Institutional Review Board of the National Center for Child Health and Development approved this study (approval number: 2025-183).

### 2.3. Statistical Analysis Plan

#### 2.3.1. Characteristics of the Patients and Concomitant ASMs

Data on the characteristics of the patients, including age, sex, and epilepsy classification (focal epilepsy, generalized epilepsy, other, or unknown), were collected from the P-MICS database. For patients in the combination therapy group, information on the ASMs used concomitantly with LEV during the assessment period was also collected. Summary statistics for the characteristics of the patients and concomitant ASMs were calculated.

#### 2.3.2. Analysis of the Retention Rate

The assessment period for the retention rate was defined as the period from the date of LEV initiation to the end date of the relevant regimen. Event was defined as the discontinuation of LEV monotherapy in the monotherapy group and the discontinuation of the LEV-containing combination regimen in the combination therapy group. Patients who did not experience an event by the end date of the relevant regimen were censored. The retention rate for each treatment group was examined using the Kaplan–Meier method. The magnitude of between-group differences was evaluated by estimating the hazard ratio (HR) and its 95% confidence interval (CI) for the monotherapy group versus the combination therapy group using Cox regression models with inverse probability of treatment weighting (IPTW). In the IPTW analysis, propensity scores were assessed using age, sex, and epilepsy classification as covariates, and each patient was weighted using the inverse of the propensity score. In the subgroup analysis limited to patients with focal epilepsy, age and sex were used as covariates.

#### 2.3.3. Analysis of the Rate of Irritability or Insomnia

The assessment period for the AE rate was defined as the period from the date of LEV initiation to the onset date of an AE during treatment with the relevant regimen. Irritability was defined as the prescription of antipsychotics (ATC classification: N05A) during the assessment period. Insomnia was defined as both a diagnosis of insomnia (ICD-10 code: G470) and prescription of hypnotics and sedatives (ATC classification: N05C) during the assessment period. Event was defined as the first occurrence of an AE. Patients who did not experience an event by the end date of the relevant regimen were censored. The AE rate in each treatment group was examined using the Kaplan–Meier method. The magnitude of between-group differences was evaluated by estimating the HR and its 95% CI for the monotherapy group versus the combination therapy group using Cox regression models with IPTW. In the IPTW analysis, propensity scores were estimated using age, sex, and epilepsy classification as covariates. Each patient was weighted by the inverse of the propensity score. In the subgroup analysis, age and sex were utilized as covariates.

In the safety analysis, among patients who experienced AEs during treatment with an LEV-containing regimen, the timing of AE onset and the proportion of patients who had LEV dose modification, including discontinuation, interruption, or dose reduction, within 2 weeks after the onset of AE were also summarized. Detailed information on the antipsychotics (ATC classification: N05A) prescribed to patients who developed irritability and on hypnotics and sedatives (ATC classification: N05C) prescribed to patients who developed insomnia was collected from the P-MICS database. Summary statistics were calculated.

Data extraction, dataset processing, and statistical analyses were performed using the SAS 9.4 TS Level 1M97 software (version 9.04.01 M9P060525M7P080520).

In accordance with the database use policy, patient counts of <3 were not reported to prevent patient identification.

## 3. Results

### 3.1. Characteristics of the Patients

As of September 2025, electronic medical record data were available for 1,447,089 patients after excluding those with missing information on age or sex. In total, 6461 patients met the inclusion criteria. After excluding 2885 patients who met the exclusion criteria, 3576 patients were included in this study. Table 1 shows the characteristics of the patients. The monotherapy group included 2936 patients, and the combination therapy group comprised 640 patients. In the combination therapy group, the numbers of ASMs including LEV were 2, 3, 4, and 5 in 541, 80, 12, and 7 patients, respectively.

In the monotherapy group, children aged ≥7 to <15 years accounted for the largest proportion of patients (48.9%), followed by toddlers aged ≥1 to <7 years (40.5%). In contrast, toddlers accounted for the largest proportion in the combination therapy group (42.2%), followed by children (36.3%). The combination therapy group was more likely to have a higher proportion of younger patients aged <1 year than the monotherapy group. The median ages were 6 (range: 0–14) years in the monotherapy group and 4 (range: 0–14) years in the combination therapy group. Regarding sex, the proportions of male patients were 51.9% in the monotherapy group and 55.8% in the combination therapy group. Male patients accounted for a slightly higher proportion in both groups, and the proportions were similar between the two groups. Epilepsy classification was most frequently unknown in both groups, accounting for >50% of patients. In both groups, the proportion of patients with focal epilepsy was higher than that of patients with generalized epilepsy. Nevertheless, the distribution of epilepsy classification was similar between the two groups.

### 3.2. Concomitant ASMs

Table 2 shows the ASMs used concomitantly with LEV during the assessment period, reported for regimens used within at least three patients in any group. Among the patients receiving two ASMs, including LEV, valproic acid (VPA) was the most common concomitant ASM (30.5%), followed by carbamazepine (CBZ) (29.4%), phenobarbital (20.3%), and fosphenytoin (6.3%). Among the patients receiving three ASMs, zonisamide + VPA (15.0%) was the most common combination, followed by phenobarbital (PB) + phenytoin (13.8%), CBZ + VPA (11.3%), clonazepam + VPA, and VPA + PB (7.5%). When three or more ASMs, including LEV, were used, combinations including VPA, zonisamide, and PB were frequently observed.

### 3.3. Retention Rate

**Figure 1:**
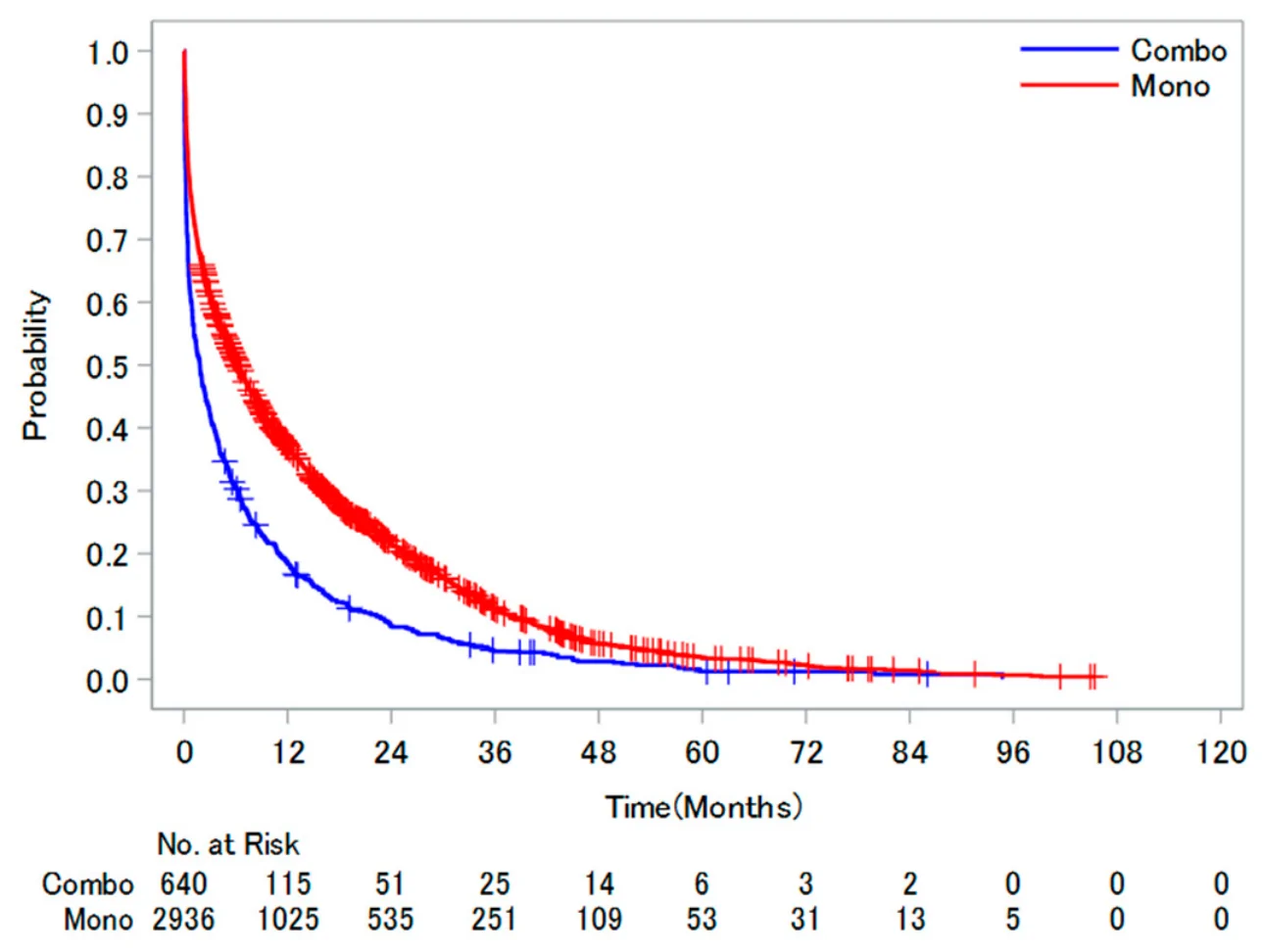
Kaplan–Meier plot of the retention rate.

Figure 1 and Table 3 show the retention rates. The median treatment durations were 180.0 (interquartile range: 30.0–545.3) days in the monotherapy group and 56.0 (interquartile range: 8.0–231.5) days in the combination therapy group. The Kaplan–Meier curves for the retention rate showed a separation between the monotherapy and combination therapy groups from the early phase after treatment initiation. In particular, the retention rate remained higher in the monotherapy group throughout the assessment period. The estimated retention rates were 50.9% versus 30.6% at 6 months, 37.2% versus 18.5% at 12 months, and 21.6% versus 8.5% at 24 months in the monotherapy and combination therapy groups, respectively. The monotherapy group had a higher retention rate than the combination therapy group at all assessment time points (adjusted HR: 0.67, 95% CI: 0.60–0.75, *p* < 0.0001).

### 3.4. Irritability

**Figure 2:**
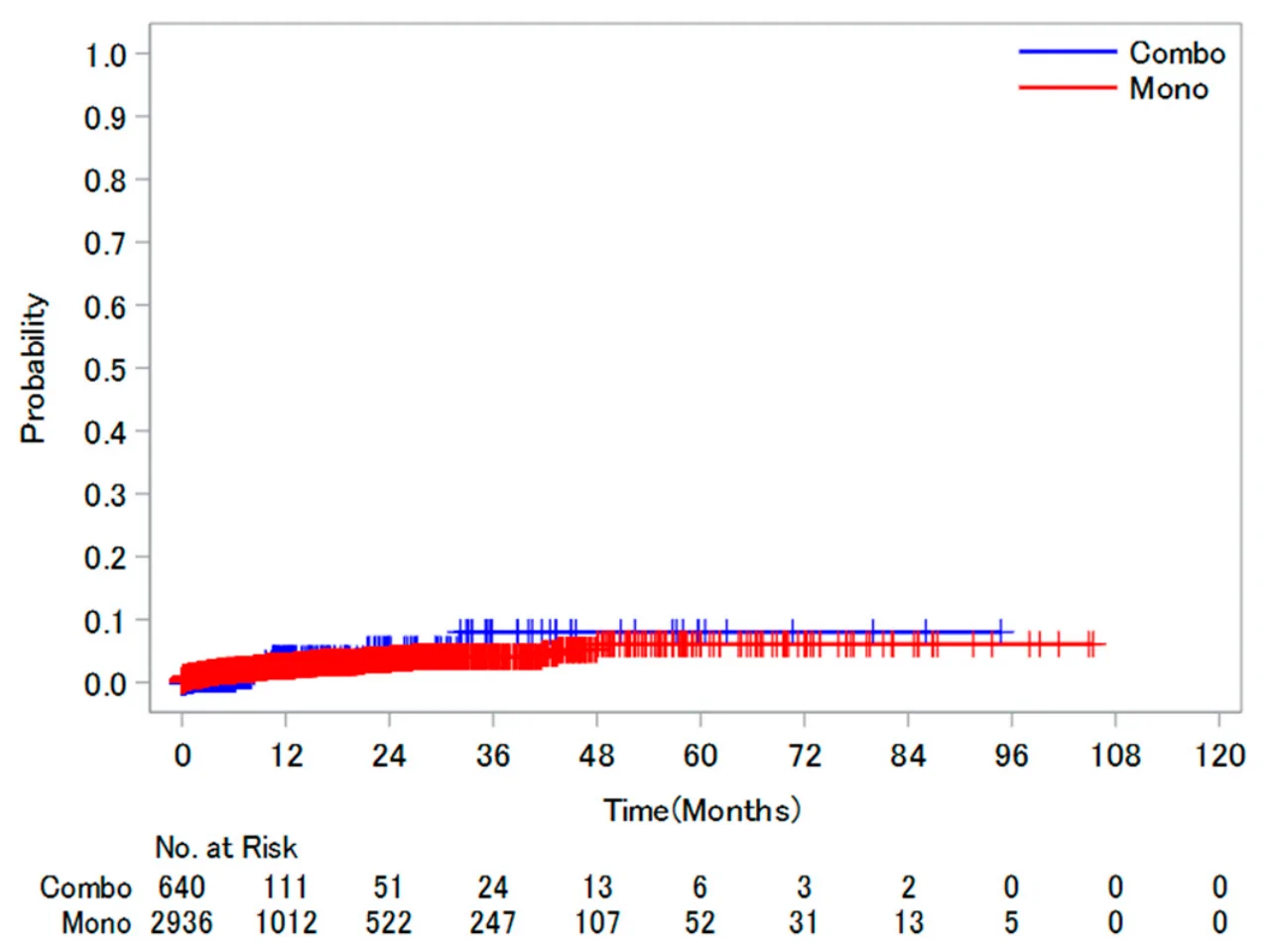
Kaplan–Meier plot of the rate of irritability.

Figure 2 and Table 4 present the rate of irritability. The Kaplan–Meier curves for the rate of irritability remained low in the monotherapy and combination therapy groups throughout the assessment period, with no clear separation between groups. The estimated rates of irritability were 1.9% versus 0.5% at 6 months, 2.5% versus 3.8% at 12 months, and 3.7% versus 5.3% at 24 months in the monotherapy and combination therapy groups, respectively. However, no significant difference was observed between the two groups (adjusted HR: 0.81, 95% CI: 0.36–1.83; *p* = 0.62).

In total, 65 (2.2%) of 2936 patients in the monotherapy group and 9 (1.4%) of 640 patients in the combination therapy group experienced irritability during treatment with an LEV-containing regimen. In both groups, irritability tended to occur more often either in the early phase after treatment initiation (<14 days) or at ≥6 months after treatment initiation. Approximately 30% of patients who developed irritability in each group had LEV dose modification within 2 weeks after onset. The antipsychotics prescribed to patients who developed irritability in both groups included risperidone, aripiprazole, and levomepromazine.

### 3.5. Insomnia

**Figure 3:**
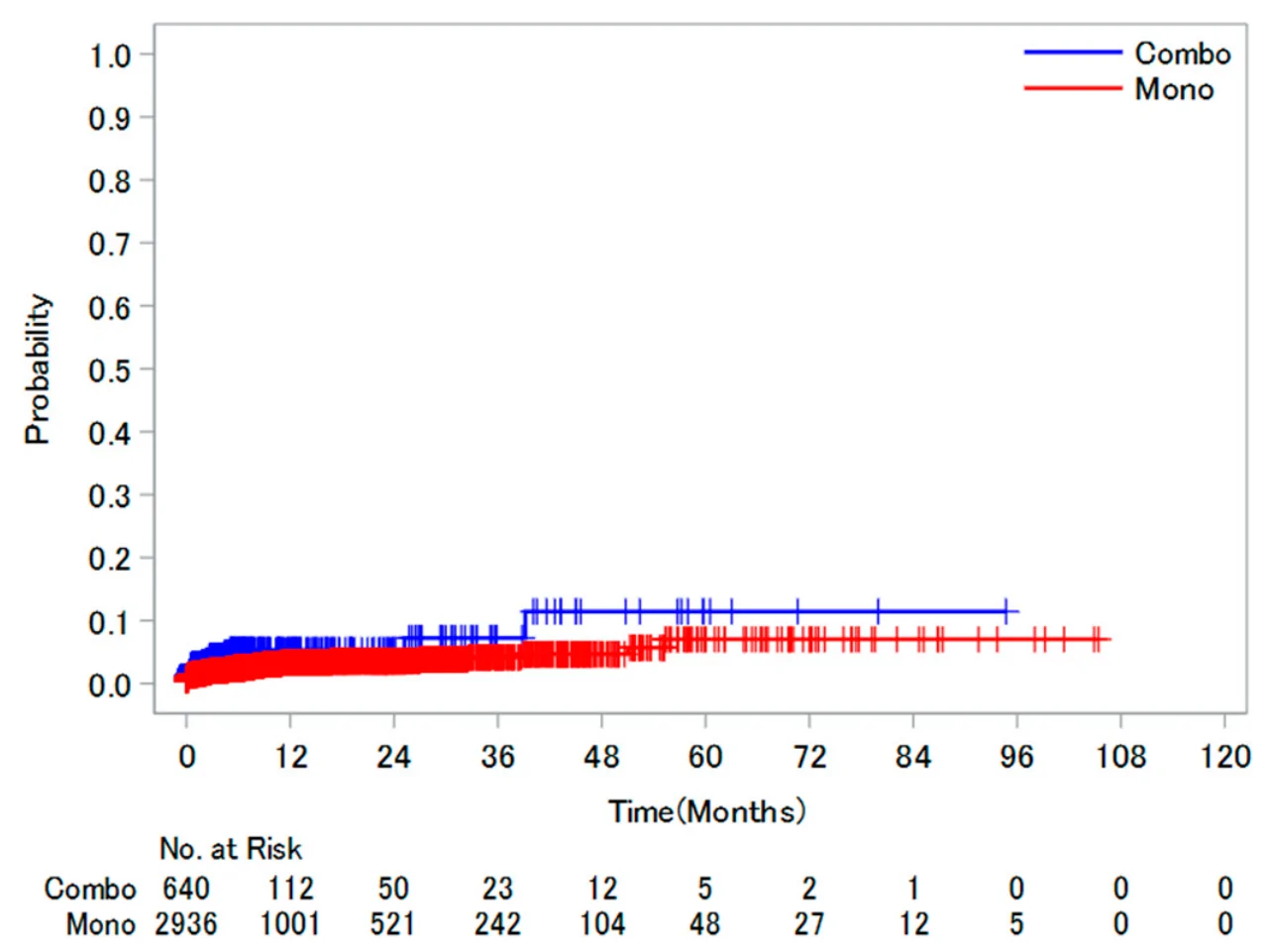
Kaplan–Meier plot of the rate of insomnia.

Figure 3 and Table 5 depict the rate of insomnia. The Kaplan–Meier curves for the rate of insomnia remained low in the monotherapy and combination therapy groups throughout the assessment period, with no clear separation between groups. The estimated rates of insomnia were 2.5% versus 5.3% at 6 months, 3.3% versus 5.3% at 12 months, and 3.5% versus 5.3% at 24 months in the monotherapy and combination therapy groups, respectively. No significant difference was observed between the two groups (adjusted HR: 0.75, 95% CI: 0.45–1.26; *p* = 0.28).

In total, 76 (2.6%) of 2936 patients in the monotherapy group and 23 (3.6%) of 640 patients in the combination therapy group experienced insomnia during treatment with an LEV-containing regimen. In both groups, insomnia frequently occurred in the early phase after treatment initiation (<14 days). Approximately 18.4% of patients in the monotherapy group and <13.0% of patients in the combination therapy group had LEV dose modification within 2 weeks after insomnia onset. The hypnotics and sedatives prescribed to patients who developed insomnia included melatonin, triclofos, and ramelteon.

### 3.6. Subgroup Analysis of Patients with Focal Epilepsy

Considering differences in pathophysiology and treatment strategies according to epilepsy classification, a subgroup analysis was conducted among patients whose epilepsy classification was recorded as focal epilepsy in the patient characteristics. The retention rate and the rate of AEs, including irritability and insomnia, were evaluated as study outcomes.

Regarding the characteristics of the patients (Table 6), children accounted for the largest proportion in the monotherapy group (51.0%), followed by toddlers (45.1%). In the combination therapy group, toddlers accounted for the largest proportion (45.8%), followed by children (37.3%). The combination therapy group had a higher proportion of younger patients aged <1 year than the monotherapy group. The median ages were 7 (range: 0–14) years in the monotherapy group and 5 (range: 0–14) years in the combination therapy group. Regarding sex, the proportions of male patients were 50.5% in the monotherapy group and 50.8% in the combination therapy group. Male patients accounted for a slightly higher proportion in both groups, and the proportions were similar between the two groups. The age and sex distributions among patients with focal epilepsy were similar to those observed in the overall population.

Table 7 shows the ASMs used concomitantly with LEV. In patients with focal epilepsy, the ASMs used in combination with LEV included CBZ, VPA, and PB. Although VPA was the most common concomitant ASM in the overall population, CBZ was the most frequently observed concomitant ASM among patients with focal epilepsy.

The median treatment durations were 253.5 (interquartile range: 72.0–739.8) days in the monotherapy group and 40.0 (interquartile range: 10.3–272.0) days in the combination therapy group. The estimated retention rates were 58.9% versus 33.8% at 6 months, 44.7% versus 20.0% at 12 months, and 30.2% versus 10.0% at 24 months in the monotherapy and combination therapy groups, respectively. Table 8 shows subgroup analysis of retention rate, irritability, and insomnia in patients with focal epilepsy. The monotherapy group had a higher retention rate than the combination therapy group at all assessment time points (adjusted HR: 0.53, 95% CI: 0.42–0.66; *p* < 0.0001). The results of the subgroup analysis of the retention rate in patients with focal epilepsy were consistent with those observed in the overall population.

The estimated rates of irritability were 1.3% versus 0% at 6 months, 1.6% versus 6.2% at 12 months, and 3.2% versus 6.2% at 24 months in the monotherapy and combination therapy groups, respectively, with no significant between-group difference (adjusted HR: 0.43, 95% CI: 0.10–1.89; *p* = 0.26). These results were consistent with those found in the overall population.

The estimated rates of insomnia were 1.3% versus 7.9% at 6 months, 2.0% versus 7.9% at 12 months, and 2.9% versus 7.9% at 24 months in the monotherapy and combination therapy groups, respectively. The monotherapy group had a lower rate of insomnia than the combination therapy group (adjusted HR: 0.33, 95% CI: 0.13–0.82; *p* < 0.05). Unlike the trend observed in the overall population, the risk of insomnia among patients with focal epilepsy was lower in the monotherapy group than in the combination therapy group.

## 4. Discussion

In this retrospective cohort study using data from the P-MICS database, the retention rate and the rate of AEs, including irritability and insomnia, were compared between LEV monotherapy and LEV-containing combination therapy in pediatric patients with epilepsy.

A major finding of this study was that the rate of irritability, a representative adverse event associated with LEV, did not significantly differ between the monotherapy group and the combination therapy group, which included patients receiving polytherapy. Due to potential drug interactions and cumulative effects on the central nervous system, polytherapy with ASMs has traditionally been considered a risk factor for psychiatric and behavioral symptoms [20]. However, the current study, which was based on real-world data from pediatric patients, showed a different trend. One possible explanation is that ASMs with reported mood-stabilizing effects, such as VPA, CBZ, and LTG, were frequently used in the combination therapy group [21]. Unexpected findings were also observed for insomnia, a relatively common adverse event associated with not only LEV but also other ASMs [13,19]. Therefore, the combination therapy group was expected to have a higher rate of insomnia because of the potential enhancement of these adverse effects. However, in this study, add-on LEV was not associated with a significant difference in the rate of insomnia between the monotherapy and combination therapy groups.

In this study, epilepsy was more prevalent in male patients than in female ones. Further, focal epilepsy was more common than generalized epilepsy, and the prevalence of epilepsy was higher in toddlers or children than in newborns or infants. These clinical characteristics were in accordance with those presented in large-scale epidemiological study of pediatric epilepsy [22]. Based on these findings, the patient information included in the P-MICS database reflects the general characteristics of patients with epilepsy and that the database is appropriate for use in retrospective research.

In the combination therapy group, 99 of 640 patients (15.5%) received three or more ASMs, and 19 of 640 patients (3.0%) received four or more ASMs. Although the additional efficacy of using four or more ASMs has been reported to be limited, the presence of such patients receiving extensive polytherapy suggests that the combination therapy group may have included a certain proportion of patients with treatment-resistant or more severe epilepsy [23]. In this study, the monotherapy group included patients who received LEV as the initial treatment. Meanwhile, the combination therapy group comprised patients who received LEV as an add-on therapy during treatment with other ASMs. Therefore, the combination therapy group was expected to include a higher proportion of patients with poorer prognosis compared with the monotherapy group, resulting in a substantial difference in treatment duration between the groups. For this reason, survival analysis was considered appropriate for evaluating the retention and AE rates because it allows between-group comparisons while preserving information on the time from LEV initiation to event occurrence, rather than comparing groups at a single time point. Survival analysis using Kaplan–Meier curves also enables visualization of the association between event occurrence and time, which may be particularly useful in studies with long follow-up periods, such as the current study. As expected, the treatment duration in the combination therapy group was approximately one-third of that in the monotherapy group. In addition, the number of patients meeting the definition of the combination therapy group was expected to be smaller than that meeting the definition of the monotherapy group. The reported frequencies of irritability and insomnia in the package insert are <3%. Hence, the number of events was expected to be small, resulting in a limited statistical power. Moreover, instead of propensity score matching, IPTW was used because it allows all patients in both the monotherapy and combination therapy groups to be included in the analysis. IPTW is a statistical method that estimates propensity scores based on patient covariates and weights each patient by the inverse of the propensity score, thereby adjusting covariate balance. This approach was considered suitable for reducing the effect of confounding factors in this observational study and appropriately estimating treatment effects [24].

The monotherapy group had a higher LEV retention rate than the combination therapy group at all assessment time points. The median treatment duration in the combination therapy group was approximately one-third of that in the monotherapy group. Further, some patients in the combination therapy group received five ASMs, indicating that this group included a certain proportion of patients with refractory epilepsy. Previous studies have reported that the 1-year retention rate of LEV in patients with epilepsy is approximately 50–60%. However, in the current study, the estimated 1-year retention rate was lower (37.2% in the monotherapy group and 18.5% in the combination therapy group) [16,17,18]. This finding may be attributed to the nature of this database study, which might have underestimated the actual retention rate, and to the definition used in the combination therapy group, in which the outcome was based on not only LEV continuation but also the continuation of the LEV-containing regimen.

This study has several limitations. First, epilepsy classification was recorded as unknown in >50% of patients. In addition, detailed information on epilepsy classification was not available from the P-MICS database. Because epilepsy classification in this study was determined based on ICD-10 codes, a certain proportion of patients were classified as “other” when they did not correspond to either focal epilepsy (ICD-10 codes: G400, G401, and G402) or generalized epilepsy (ICD-10 codes: G403 and G404). Because the pathophysiology and treatment strategies for epilepsy differ according to epilepsy classification, it is preferable to evaluate the retention and AE rates according to epilepsy classification. Considering differences in treatment strategies according to epilepsy classification, VPA is generally used more frequently for generalized epilepsy, whereas LEV is commonly used for focal epilepsy. Therefore, an exploratory subgroup analysis of patients with focal epilepsy was considered relevant for evaluating the real-world use of LEV in this study. Because a sufficient number of patients with focal epilepsy were available for subgroup analysis, this population was selected for further analysis. In this subgroup analysis, the monotherapy group had a higher retention rate than the combination therapy group, and no significant difference was observed between the two groups in terms of the rate of irritability. These findings were consistent with those observed in the overall population. In contrast, the monotherapy group had a lower rate of insomnia than the combination therapy group, showing a trend different from that observed in the overall population. Because the number of events in this subgroup analysis was limited, interpretation of these results is constrained. The subgroup analysis was exploratory, and further studies must be performed. Second, treatment efficacy, particularly seizure control, could not be directly evaluated. Because this was an observational study, seizure frequency, which is an indicator of seizure control, could not be examined. Therefore, the retention rate was used as an indirect indicator reflecting long-term efficacy and tolerability. The retention rate does not directly demonstrate efficacy. However, it is useful for understanding the clinical utility of treatment in real-world practice. In epilepsy, which generally requires long-term treatment, evaluation of the retention rate is clinically significant because treatment selection should consider the quality of life of patients. Third, the rate of irritability or insomnia could not be directly evaluated. As this was an observational study, AEs could not be prospectively assessed. As surrogate measures, irritability was defined as the prescription of antipsychotics (ATC classification: N05A) during the assessment period. Insomnia was defined as a prescription of hypnotics and sedatives (ATC classification: N05C) and a diagnosis of insomnia (ICD-10 code: G470) during the assessment period. Therefore, caution is required when interpreting the results. Fourth, because of the characteristics of the database, complete information on medication use could not be obtained. Treatment continuation in this study was evaluated based on prescription records. Therefore, outpatient prescriptions, dose titration or tapering, and short gaps between prescriptions might not have been completely captured. Consequently, some patients who actually continued their treatment with an LEV-containing regimen might have been evaluated as having discontinued the regimen, which could have contributed to the lower retention rate observed in this study. Fifth, because this was a database study, not all covariates could be examined. The characteristics of the patients between groups were adjusted using IPTW with age, sex, and epilepsy classification used as covariates. However, we could not evaluate or adjust for factors that could not be sufficiently obtained from the P-MICS database, such as seizure frequency, epilepsy severity, history of status epilepticus, underlying diseases, developmental status, detailed seizure classification, etiology, and the background of ASM selection. In addition, although newborns were included in this study to broadly assess the real-world use of LEV, neonatal epilepsy may differ from epilepsy in infants and older children in terms of seizure classification, etiology, ASM selection, and treatment strategies. Therefore, residual differences in disease severity and treatment background, including clinical characteristics specific to newborns that could not be sufficiently evaluated or adjusted for, might have remained between the monotherapy and combination therapy groups, and the results should be interpreted with caution.

Despite these limitations, this study used real-world data collected from pediatric medical institutions across Japan and followed >3500 pediatric patients over a 9-year period. In the pediatric field, where clinical data remain limited, the findings on LEV use, retention rate, and AE rate, including irritability and insomnia, are considered valuable and clinically significant.

## 5. Conclusions

In this retrospective cohort study using data from the P-MICS database, the retention rate and the rate of irritability or insomnia between LEV monotherapy and LEV-containing combination therapy in pediatric patients with epilepsy were compared. The monotherapy group had a higher retention rate than the combination therapy group. Meanwhile, the rates of irritability or insomnia did not significantly differ between the two groups. Based on these findings, adding LEV to other ASMs was not associated with an evident increase in the rate of irritability or insomnia. Although ASM polytherapy has traditionally been considered to increase the risk of psychiatric and behavioral symptoms, the current study showed a different trend. These findings may provide useful real-world information when considering LEV-containing treatment regimens for pediatric patients with epilepsy aged 0–15 years, particularly with regard to long-term treatment retention and monitoring for behavioral symptoms and sleep-related adverse events.

## Figures and Tables

**Table 1:** Characteristics of the patients.

	Monotherapy Group	Combination Therapy Group				
No. of ASMs	1	2	3	4	5	Total
	*n* (%)	*n* (%)	*n* (%)	*n* (%)	*n* (%)	*n* (%)
No. of Patients	2936	541	80	12	7	640
Age, years						
Newborn (<4 weeks)	32 (1.1)	38 (7.0)	5 (6.3)	0 (0.0)	0 (0.0)	43 (6.7)
Infant (4 weeks to <1 year)	277 (9.4)	76 (14.0)	15 (18.8)	<5 (41.7)	0 (0.0)	95 (14.8)
Toddler (1 to <7 years)	1190 (40.5)	217 (40.1)	41 (51.3)	7 (58.3)	<7 (100.0)	270 (42.2)
Child (7 to <15 years)	1437 (48.9)	210 (38.8)	19 (23.8)	<3 (25.0)	<3 (42.9)	232 (36.3)
Median	6	5	3	1	4	4
Range	0–14	0–14	0–14	0–7	1–9	0–14
Sex						
Male	1525 (51.9)	296 (54.7)	46 (57.5)	10 (83.3)	5 (71.4)	357 (55.8)
Female	1411 (48.1)	245 (45.3)	34 (42.5)	<3 (25.0)	<3 (42.9)	283 (44.2)
Epilepsy classification ^1^						
Focal	614 (20.9)	100 (18.5)	14 (17.5)	4 (33.3)	0 (0.0)	118 (18.4)
Generalized	81 (2.8)	30 (5.5)	14 (17.5)	<3 (25.0)	<3 (42.9)	47 (7.3)
Other	646 (22.0)	98 (18.1)	26 (32.5)	4 (33.3)	<3 (42.9)	130 (20.3)
Unknown	1595 (54.3)	313 (57.9)	26 (32.5)	<3 (25.0)	4 (57.1)	345 (53.9)

^1^ In the epilepsy classification, “other” refers to epilepsy other than focal or generalized epilepsy, and “unknown” indicates that the type of epilepsy was not recorded. The percentages were calculated using the number of patients in each column as the denominator. ASM, anti-seizure medication.

**Table 2:** ASMs concomitantly used with LEV during the assessment period (observed in ≥3 patients).

	Combination Therapy Group				
No. of ASMs	2	3	4	5	Total
	*n* (%)	*n* (%)	*n* (%)	*n* (%)	*n* (%)
No. of Patients	541	80	12	7	640
Concomitant ASMs					
VPA	165 (30.5)	-	-	-	165 (25.8)
CBZ	159 (29.4)	-	-	-	159 (24.8)
PB	110 (20.3)	-	-	-	110 (17.2)
PHT	34 (6.3)	-	-	-	34 (5.3)
LCM	21 (3.9)	-	-	-	21 (3.3)
CZP	17 (3.1)	-	-	-	17 (2.7)
ZNS + VPA	-	12 (15.0)	-	-	12 (1.9)
ZNS	12 (2.2)	-	-	-	12 (1.9)
PB + PHT	-	11 (13.8)	-	-	11 (1.7)
CBZ + VPA	-	9 (11.3)	-	-	9 (1.4)
LTG	9 (1.7)	-	-	-	9 (1.4)
CZP + VPA	-	6 (7.5)	-	-	6 (0.9)
VPA + PB	-	6 (7.5)	-	-	6 (0.9)
CBZ + PB	-	4 (5.0)	-	-	4 (0.6)
TPM	4 (0.7)	-	-	-	4 (0.6)
CBZ + ZNS	-	3 (3.8)	-	-	3 (0.5)
CZP + ZNS + VPA + PB	-	-	-	3 (42.9)	3 (0.5)
ZNS + VPA + PB	-	-	3 (25.0)	-	3 (0.5)
TPM + VPA	-	3 (3.8)	-	-	3 (0.5)
PER + LCM	-	3 (3.8)	-	-	3 (0.5)
PER	3 (0.6)	-	-	-	3 (0.5)

The number of ASMs includes LEV. A dash indicates that the corresponding concomitant ASM or ASM combination was observed in fewer than three patients or was not observed in the treatment group. ASM, anti-seizure medication; CBZ, carbamazepine; CZP, clonazepam; LCM, lacosamide; LEV, levetiracetam; LTG, lamotrigine; PB, phenobarbital; PER, perampanel; PHT, phenytoin; TPM, topiramate; VPA, valproic acid; ZNS, zonisamide.

**Table 3:** Analysis of the retention rate.

	Monotherapy Group	Combination Therapy Group (Total)
	*n*	*n*
No. of Patients	2936	640
No. of events	2679	623
vs. combination therapy (total)		
Crude HR (95% CI)	0.66 (0.59–0.73)	
*p*-value	<0.0001	
Adjusted HR ^1^ (95% CI)	0.67 (0.60–0.75)	
*p*-value	<0.0001	

^1^ The adjusted HR was estimated using IPTW and adjusted for age, sex, and epilepsy classification. The HRs for monotherapy relative to combination therapy were calculated. Events were defined as the discontinuation of the LEV-containing regimen. CI, confidence interval; HR, hazard ratio; IPTW, inverse probability of treatment weighting; LEV, levetiracetam.

**Table 4:** Analysis and characteristics of irritability during LEV treatment.

	Monotherapy Group	Combination Therapy Group (Total)
	*n*	*n*
No. of Patients	2936	640
No. of events	65	9
vs. combination therapy (total)		
Crude HR (95% CI)	0.82 (0.38–1.77)	
*p*-value	0.62	
Adjusted HR ^1^ (95% CI)	0.81 (0.36–1.83)	
*p*-value	0.62	
	***n* (%)**	***n* (%)**
**No. of Patients with Irritability**	**65**	**9**
Time to onset		
<14 days	21 (32.3)	<3 (33.3)
≥14 days, <1 month	5 (7.7)	0 (0.0)
≥1 month, <3 months	9 (13.8)	<3 (33.3)
≥3 months, <6 months	8 (12.3)	0 (0.0)
≥6 months, <12 months	8 (12.3)	5 (55.6)
≥12 months	14 (21.5)	<3 (33.3)
LEV dose modification		
Any dose modification	18 (27.7)	<3 (33.3)
Discontinuation	<3 (4.6)	0 (0.0)
Interruption	3 (4.6)	<3 (33.3)
Dose reduction	13 (20.0)	<3 (33.3)
Prescribed antipsychotics		
Risperidone	27 (41.5)	5 (55.6)
Aripiprazole	11 (16.9)	<3 (33.3)
Levomepromazine	4 (6.2)	<3 (33.3)
Quetiapine	5 (7.7)	0 (0.0)
Quetiapine + risperidone	3 (4.6)	0 (0.0)
Haloperidol	3 (4.6)	0 (0.0)
Haloperidol + risperidone	3 (4.6)	0 (0.0)

^1^ The adjusted HR was estimated using IPTW and adjusted for age, sex, and epilepsy classification. The HRs for monotherapy relative to combination therapy were calculated. Events were defined as irritability during treatment with the LEV-containing regimen. The percentages in the sections on time to onset, LEV dose modification, and antipsychotics were calculated using the number of patients with irritability in each group as the denominator. LEV dose modification was defined as discontinuation, interruption, or dose reduction within 2 weeks after the onset of irritability. CI, confidence interval; HR, hazard ratio; IPTW, inverse probability of treatment weighting; LEV, levetiracetam.

**Table 5:** Analysis and characteristics of insomnia during LEV treatment.

	Monotherapy Group	Combination Therapy Group (Total)
	*n*	*n*
No. of Patients	2936	640
No. of events	76	23
vs. combination therapy (total)		
Crude HR (95% CI)	0.72 (0.43–1.20)	
*p*-value	0.20	
Adjusted HR ^1^ (95% CI)	0.75 (0.45–1.26)	
*p*-value	0.28	
	***n* (%)**	***n* (%)**
**No. of Patients with Insomnia**	**76**	**23**
Time to onset		
<14 days	36 (47.4)	9 (39.1)
≥14 days, <1 month	4 (5.3)	6 (26.1)
≥1 month, <3 months	11 (14.5)	4 (17.4)
≥3 months, <6 months	7 (9.2)	<3 (13.0)
≥6 months, <12 months	10 (13.2)	0 (0.0)
≥12 months	8 (10.5)	<3 (13.0)
LEV dose modification		
Any dose modification	14 (18.4)	<3 (13.0)
Discontinuation	<3 (3.9)	0 (0.0)
Interruption	<3 (3.9)	0 (0.0)
Dose reduction	12 (15.8)	<3 (13.0)
Prescribed hypnotics and sedatives		
Melatonin	11 (14.5)	<3 (13.0)
Triclofos	3 (3.9)	6 (26.1)
Ramelteon	7 (9.2)	<3 (13.0)
Dexmedetomidine + triclofos + midazolam	3 (3.9)	3 (13.0)
Dexmedetomidine + triclofos + midazolam + chloral hydrate	6 (7.9)	0 (0.0)
Triclofos + midazolam	3 (3.9)	<3 (13.0)
Triclofos + ramelteon	3 (3.9)	<3 (13.0)
Dexmedetomidine + triclofos + Midazolam + ramelteon	3 (3.9)	0 (0.0)
Dexmedetomidine + triclofos + midazolam + ramelteon + chloral hydrate	3 (3.9)	0 (0.0)
Dexmedetomidine + midazolam	3 (3.9)	0 (0.0)
Triclofos + chloral hydrate	<3 (3.9)	<3 (13.0)

^1^ The adjusted HR was estimated using IPTW and adjusted for age, sex, and epilepsy classification. The HRs for monotherapy relative to combination therapy were calculated. Events were defined as insomnia, operationalized as both a diagnosis of insomnia and a prescription of hypnotics and sedatives during treatment with an LEV-containing regimen. The percentages in the sections on time to onset, LEV dose modification, and hypnotics and sedatives were calculated using the number of patients with insomnia in each group as the denominator. LEV dose modification was defined as discontinuation, interruption, or dose reduction within 2 weeks after insomnia onset. CI, confidence interval; HR, hazard ratio; IPTW, inverse probability of treatment weighting; LEV, levetiracetam.

**Table 6:** Characteristics of patients with focal epilepsy in the subgroup analysis.

	Monotherapy Group	Combination Therapy Group			
No. of ASMs	1	2	3	4	Total
	*n* (%)	*n* (%)	*n* (%)	*n* (%)	*n* (%)
No. of Patients	614	100	14	4	118
Age, years					
Newborn (<4 weeks)	<3 (0.5)	3 (3.0)	0 (0.0)	0 (0.0)	3 (2.5)
Infant (4 weeks to <1 year)	<25 (4.1)	14 (14.0)	<3 (21.4)	<3 (75.0)	17 (14.4)
Toddler (1 to <7 years)	277 (45.1)	45 (45.0)	<10 (71.4)	<3 (75.0)	54 (45.8)
Child (7 to <15 years)	313 (51.0)	38 (38.0)	<10 (71.4)	0 (0.0)	44 (37.3)
Median	7	5	6	1	5
Range	0–14	0–14	0–12	0–4	0–14
Sex					
Male	310 (50.5)	52 (52.0)	4 (28.6)	4 (100.0)	60 (50.8)
Female	304 (49.5)	48 (48.0)	10 (71.4)	0 (0.0)	58 (49.2)

The percentages were calculated using the number of patients in each column as the denominator. ASM, anti-seizure medication.

**Table 7:** ASMs concomitantly used with LEV during the assessment period (observed in ≥3 patients with focal epilepsy).

	Combination Therapy Group (Total)
	*n* (%)
No. of Patients	118
Concomitant ASMs	
CBZ	51 (43.2)
VPA	21 (17.8)
PB	10 (8.5)
LCM	7 (5.9)
PHT	3 (2.5)

ASM, anti-seizure medication; CBZ, carbamazepine; LCM, lacosamide; LEV, levetiracetam; PB, phenobarbital; PHT, phenytoin; VPA, valproic acid.

**Table 8:** Subgroup analysis of retention rate, irritability, and insomnia in patients with focal epilepsy.

	Monotherapy Group	Combination Therapy Group (Total)
	*n*	*n*
No. of Patients	614	118
No. of events (discontinuation of the LEV-containing regimen)	546	113
vs. combination therapy (total)		
Crude HR (95% CI)	0.53 (0.42–0.66)	
*p*-value	<0.0001	
Adjusted HR ^1^ (95% CI)	0.53 (0.42–0.66)	
*p*-value	<0.0001	
No. of events (irritability)	13	3
vs. combination therapy (total)		
Crude HR (95% CI)	0.44 (0.10–1.86)	
*p*-value	0.26	
Adjusted HR ^1^ (95% CI)	0.43 (0.10–1.89)	
*p*-value	0.26	
No. of events (insomnia)	12	7
vs. combination therapy (total)		
Crude HR (95% CI)	0.30 (0.11–0.77)	
*p*-value	<0.05	
Adjusted HR ^1^ (95% CI)	0.33 (0.13–0.82)	
*p*-value	<0.05	

^1^ The adjusted HR was estimated using IPTW and adjusted for age and sex. The HRs for monotherapy relative to combination therapy were calculated. For the retention rate, events were defined as discontinuation of the LEV-containing regimen. For irritability, events were defined as prescriptions of antipsychotics during treatment with an LEV-containing regimen. For insomnia, events were defined as both a diagnosis of insomnia and a prescription of hypnotics and sedatives during treatment with an LEV-containing regimen. CI, confidence interval; HR, hazard ratio; IPTW, inverse probability of treatment weighting; LEV, levetiracetam.

## Data Availability

The data presented in this study are available upon request from the corresponding author. The data are not publicly available due to the protection of personal information.

## Supplementary Materials

The following supporting information can be downloaded at https://www.mdpi.com/article/10.3390/children13080997/s1, Table S1: List of rescue medications (excluding injectable formulations); Table S2: List of rescue medications (injectables).

## Abbreviations

The following abbreviations are used in this manuscript:

AE	Adverse event
ASM	Anti-seizure medication
ATC	Anatomic Therapeutic Chemical
CBZ	Carbamazepine
CI	Confidence interval
CZP	Clonazepam
HR	Hazard ratio
ICD-10	International Classification of Diseases, 10th Revision
IPTW	Inverse probability of treatment weighting
LCM	Lacosamide
LEV	Levetiracetam
LTG	Lamotrigine
PB	Phenobarbital
PER	Perampanel
PHT	Phenytoin
P-MICS	Pediatric Medical Information Collection System
TPM	Topiramate
VPA	Valproic acid
ZNS	Zonisamide
