# Supplementary material for: Long-Term Follow-Up of Levetiracetam Monotherapy Versus Add-On Therapy in Pediatric Epilepsy
Source: Children (Basel). 2026 Jul 28;13(8):997. doi: 10.3390/children13080997 (PMC13511238; doi:10.3390/children13080997)
Supplement: Supplementary file 1 [file children-13-00997-s001.zip › children-4442574-supplementary.pdf]

Supplementary Table S1. List of rescue medication (excluding injectable formulations)

Generic Name	Brand Name	ATC Classification	Individual Drug Code
Diazepam	Diup Suppository 4 mg	N05BA01	1124701J1022
	Diup Suppository 6 mg		1124701J2029
	Diup Suppository 10 mg		1124701J3025
Chloral Hydrate	Escle Suppository 250 mg	N05CC01	1123700J1020
	Escle Suppository 500 mg		1123700J2027
	Escle Rectal Kit 500 mg		1123700X1023
Phenobarbital	Wacobital Suppository 15 mg	N03AA02	1125700J1025
	Wacobital Suppository 30 mg		1125700J3028
	Wacobital Suppository 50 mg		1125700J4032
	Wacobital Suppository 100 mg		1125700J5039
	Rupial Suppository 25 mg		1125700J2021
	Rupial Suppository 50 mg		1125700J4024
	Rupial Suppository 100 mg		1125700J5020
	Phenobal Elixir 0.4%		1125004S1030
Midazolam	Buccolam Oromucosal Solution 2.5mg	N05CD08	1139700Q1029
	Buccolam Oromucosal Solution 5mg		1139700Q2025
	Buccolam Oromucosal Solution 7.5mg		1139700Q3021
	Buccolam Oromucosal Solution 10mg		1139700Q4028
Fenfluramine Hydrochloride	Fintepla Oral Solution 2.2mg/mL	N03AX26	1139016S1020

Supplementary Table S2. List of rescue medication (injectables)

Generic Name	Brand Name	ATC Classification	Individual Drug Code
Diazepam	Horizon Injection 10mg	N05BA01	1124402A2045
	Cercine Injection 5mg		1124402A1030
	Cercine Injection 10mg		1124402A2053
	Diazepam Injection 5mg		1124402A1014
	Diazepam Injection 5mg (TAIYO)		1124402A1049
	Diazepam Injection 5mg (NIG)		1124402A1057
	Diazepam Injection 10mg		1124402A2010
	Diazepam Injection 10mg (TAIYO)		1124402A2061
	Diazepam Injection 10mg (NIG)		1124402A2070
Perampanel Hydrate	Fycompa Intravenous Infusion 2mg	N03AX22	1139405D1025
Midazolam	Dormicum Injection 10mg	N05CD08	1124401A1052
	Midfresa IV Injection 0.1%		1139401A1020
	Midazolam Injection 10 mg/2 mL		1124401A1010
	Midazolam Injection 10 mg (TAIYO)		1124401A1079
	Midazolam Injection 10 mg (TEVA)		1124401A1087
	Midazolam Injection 10 mg (SANDOZ)		1124401A1060
	Midazolam Injection 10 mg (NIG)		1124401A1095
Lorazepam	Lorapita® IV Injection 2 mg	N05BA06	1139403A1020
Fosphenytoin	Hostoin® IV Injection 750 mg	N03AB05	1132401A1020
Lacosamide	Vimpat® Intravenous Infusion 100 mg	N03AX18	1139404A2020
	Vimpat® Intravenous Infusion 200 mg		1139404A1024
Phenobarbital	Nobelbar® IV Injection 250 mg	N03AA02	1139400F1023
	Phenobarbital Injection 100 mg		1125402A1054
	Phenobarbital Injection 10% (Nobel)		1125402A1046
Phenytoin Sodium	Aleviatin® Injection 250 mg	N03AB02	1132400A1033
Brivaracetam	Briviact® IV Injection 25 mg	N03AX23	1139406A1023

Levetiracetam	Keppra® Intravenous Infusion 500 mg	N03AX14	1139402A1025
	Levetiracetam Intravenous Infusion 500 mg (Nisshin)		1139402A2030
	Levetiracetam Intravenous Infusion 500 mg (Meiji)		1139402A2048
	Levetiracetam Intravenous Infusion 500 mg (Amel)		1139402A2021
